# Brain Activation During Virtual Reality Symptom Provocation in Obsessive-Compulsive Disorder: Proof-of-Concept Study

**DOI:** 10.2196/47468

**Published:** 2024-01-19

**Authors:** Martine J van Bennekom, Guido van Wingen, Willem Benjamin Bruin, Judy Luigjes, Damiaan Denys

**Affiliations:** 1 Adult Psychiatry Amsterdam UMC location University of Amsterdam Amsterdam Netherlands

**Keywords:** virtual reality, obsessive-compulsive disorder, VR, symptom provocation, MRI, neuroimaging, OCD

## Abstract

**Background:**

Obsessive-compulsive disorder (OCD) is a psychiatric disorder characterized by obsessions and compulsions. We previously showed that a virtual reality (VR) game can be used to provoke and measure anxiety and compulsions in patients with OCD. Here, we investigated whether this VR game activates brain regions associated with symptom provocation.

**Objective:**

In this study, we aim to investigate the neural regions that are activated in patients with OCD when they are interactively confronted with a symptom-provoking event and when they are performing compulsive actions in VR.

**Methods:**

In a proof-of-concept study, we investigated brain activation in response to the VR game in 9 patients with OCD and 9 healthy controls. Participants played the VR game while regional changes in blood oxygenation were measured using functional magnetic resonance imaging. We investigated brain activation in relation to OCD-related events and virtual compulsions in the VR game. Due to low statistical power because of the sample size, we also reported results at trend significance level with a threshold of *P*<.10. Additionally, we investigated correlations between OCD severity and brain activation.

**Results:**

We observed a trend for increased activation in the left amygdala (*P*=.07) upon confrontation with OCD-related events and for increased activation in the bilateral amygdala (*P*=.06 and *P*=.09) and right insula (*P*=.09) when performing virtual compulsive actions in patients with OCD compared to healthy controls, but this did not attain statistical significance. The amygdala and insula activation did not correlate with OCD severity.

**Conclusions:**

The findings of this proof-of-concept study indicate that VR elicits brain activation in line with previous provocation studies. Our findings need to be replicated in a study with a larger sample size. VR may be used as an innovative and unique method of interactive symptom provocation in future neuroimaging studies.

**Trial Registration:**

Netherlands Trial Register NTR6420; https://onderzoekmetmensen.nl/nl/trial/25755

## Introduction

Obsessive-compulsive disorder (OCD) is a chronic, debilitating disorder characterized by obsessions, recurring involuntary thoughts that are frequently linked to compulsions—mental or physical acts to control provoked emotions of fear or restlessness. The obsessions and compulsions are often accompanied by feelings of anxiety and uncertainty and cause a high level of suffering [1]. OCD has a 2%-3% lifetime prevalence and is associated with significant impairment in social and occupational functioning [2].

An OCD diagnosis is usually based on an interpretation of clinical signs and symptoms as retrospectively expressed by the patient. Assessment by a clinician while patients are actually experiencing symptoms in the consulting room may provide a more realistic image of the symptoms and improve the diagnostic process. This can be achieved by symptom provocation [3].

Virtual reality (VR) is one way of achieving symptom provocation in patients with OCD. There are numerous examples of studies that have investigated the use of VR to provoke OCD symptoms in order to improve an OCD diagnosis or provide targeted treatment. For example, Laforest et al [4] showed that exposure to a virtual contaminated toilet in a VR immersion chamber led to an increase in anxiety and heart rate in patients with OCD with contamination fear compared to that in healthy controls.

Furthermore, in a systematic review and meta-analysis by Dehghan et al [5], it was found that VR environments were capable of significantly increasing anxiety, disgust, uncertainty, washing urges, time spent on checking, and the number of checks in patients with OCD compared to healthy controls.

**Figure 1:**
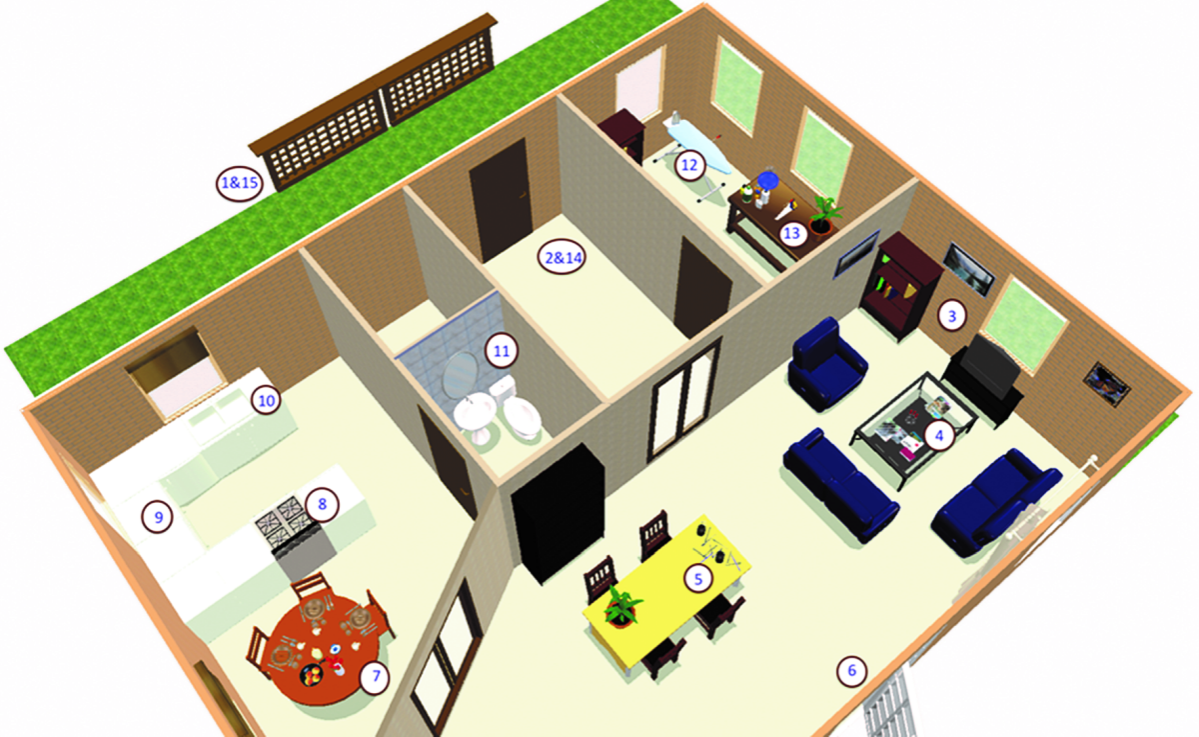
A 3D map of the house indicating obsessive-compulsive disorder–related items. 1: locking the gate (start); 2: locking the front door (start); 3: switching off the television; 4: extinguishing the candle; 5: organizing pencils; 6: closing the window; 7: cleaning the breakfast table; 8: turning off the gas stove; 9: organizing the cans; 10: cleaning the sink; 11: hand-washing after using the toilet; 12: switching off the flat iron; 13: organizing hazardous substances; 14: locking the front door (end); and 15: locking the gate (end).

**Figure 2:**
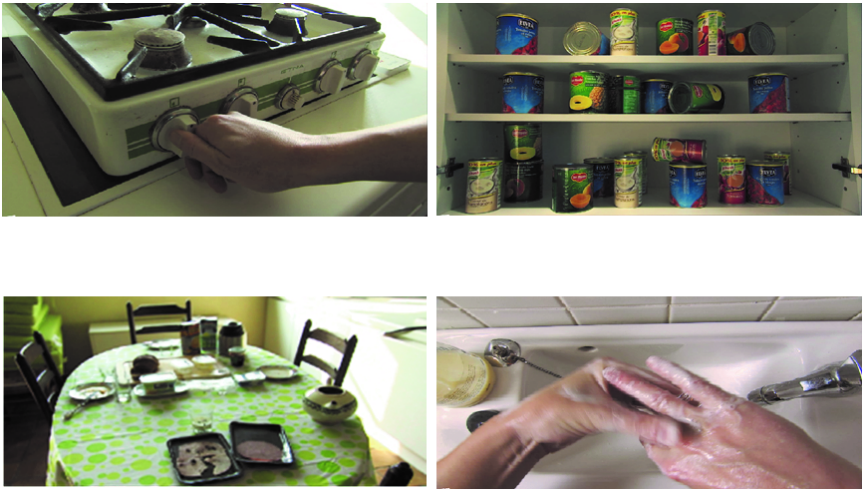
Screenshots from the virtual reality game.

In 2 former studies, we investigated an interactive VR game designed to provoke and assess OCD symptoms in a controlled and standardized way [6,7]. Figures 1 and 2 show a schematic outline and screenshots from the VR game. The VR game is designed to actively confront patients with OCD-related events in a standard household environment. It is a first-person–perspective game composed of video images of an actual house. Patients are asked to carefully check the house, which is left behind in a hurry by a friend. They walk through the house in a preset order and are confronted with 15 OCD-related events (eg, turning off the gas stove). Patients are asked to solve these events and subsequently check or repeat the events as often as desired (for full details, see van Bennekom et al [7]). We showed that this VR game, when played on a laptop screen, was able to provoke higher levels of anxiety and virtual compulsions in patients with OCD than in healthy controls [6,7].

In this study, we modified the VR game to enable performance inside a functional magnetic resonance imaging (fMRI) scanner. In contrast to traditional “passive” fMRI symptom provocation tasks mostly using images, written verbal stimuli, emotional faces, or neurocognitive tasks with emotional interference [8,9], this VR game is interactive and realistic and therefore actively immerses patients whilst inside the scanner. This allows us to gain insight in to blood oxygenation level–dependent (BOLD) derived brain activation while patients are confronted with OCD-related events and while they perform virtual compulsive actions. To our knowledge, this is the first study using a VR game with fMRI for symptom provocation in OCD.

Recent meta-analyses of fMRI, positron emission tomography, and single-photon emission computed tomography studies underline several brain regions involved in the pathophysiology of OCD. Abnormalities in cortico-striato-thalamo-cortical pathways—circuits connecting the cortex, striatum, basal ganglia, and thalamus—are involved in the pathophysiological substrate [10]. In a recent meta-analysis of fMRI, positron emission tomography, and single-photon emission computed tomography studies conducted by Thorsen et al [9], brain activation during symptom provocation was compared between patients with OCD and healthy controls. They found higher levels of activation in the bilateral amygdala, right putamen, orbitofrontal cortex (OFC) extending into the anterior cingulate cortex (ACC) and ventromedial prefrontal cortex, and middle temporal and left inferior occipital cortices during emotional processing in patients than in healthy controls. In patients with OCD with a higher rate of comorbidity with anxiety or mood disorders, they found more pronounced activation in the right putamen, amygdala, and insula. Another preceding smaller meta-analysis of neuroimaging symptom provocation studies in OCD, conducted by Rotge et al [11], also found an increased likelihood of activation in 19 clusters in patients with OCD compared to healthy controls. These included the OFC, ACC, precuneus, and thalamus. Although paradigms have been developed to induce the urge to check in patients with OCD [12], to our knowledge, no provocation procedures to induce actual checking behavior have been applied in fMRI studies before.

Because our VR game represents a new and innovative technique for fMRI symptom provocation, we decided to perform a proof-of-concept study with a limited sample size. In this study, we aim to investigate the neural regions that are activated in patients with OCD when they are interactively confronted with a symptom-provoking event and when they are performing compulsive actions in VR. Moreover, we aim to investigate whether activation in these regions is related to OCD symptom severity. We hypothesized that (1) playing the VR game inside a fMRI scanner would lead to increased brain activity within the OFC, ACC, amygdala, right putamen, and right insula in patients with OCD compared to that in healthy controls, and (2) a positive correlation exists between the degree of brain activation and the severity of OCD in patients.

## Methods

### Participants

We recruited 9 patients with OCD from December 2017 to March 2020 at the Psychiatric Outpatient Department of Amsterdam University Medical Center by means of information letters provided by their treating clinicians. Patients were also recruited through the Dutch OCD website Dwang.eu [13]. This sample size is in line with recommendations for proof-of-concept fMRI studies [14]. All included patients had a primary diagnosis of OCD, as determined by a psychiatrist and confirmed by the Mini-International Neuropsychiatric Interview in accordance with the DSM-IV (Diagnostic and Statistical Manual of Mental Disorders, Fourth Edition) criteria [15]. We aimed to assess a clinically relevant group of patients with OCD including those with (mild to moderate) comorbid psychiatric disorders, under the condition that OCD was the primary diagnosis. We recruited 9 age- and gender-matched healthy controls through advertisements at the Amsterdam University Medical Center and by emailing individuals who formerly participated in research projects at our department. The healthy controls were free of any current mental disorders, as validated with the Mini-International Neuropsychiatric Interview. We excluded subjects with a history of severe neurological or cardiovascular disorders, psychotic disorder, bipolar disorder, intellectual disability, and alcohol or substance abuse during the last 6 months. Furthermore, the use of medication potentially influencing cerebral blood flow, uncorrected hearing or vision problems, and irregular sleep/wake rhythm were exclusion criteria, as well as other contraindications for scanning with a magnetic resonance imaging (MRI) scanner.

### Ethical Considerations

The study was approved by the Medical Ethics Committee of the Academic Medical Center of the University of Amsterdam (case number NL59652.018.16). All participants provided written informed consent before enrollment.

### Procedure

The procedure of fMRI scanning was carried out at the Spinoza Centre for Neuroimaging, Amsterdam, the Netherlands. On the test day, trained clinical researchers obtained clinical and demographic data using questionnaires. After that, participants practiced controlling the VR game through manual button boxes in a mock scanner. For baseline measurements, they first watched a calming movie with nature scenes inside the scanner. Finally, they played the VR game during an fMRI scanning session. Trained technicians at the Spinoza Centre for Neuroimaging performed the scanning procedure in the presence of a trained researcher.

### Patient and Public Involvement

Patients or the public were not involved in the design, conduct, reporting, or dissemination plans of our research.

### Assessments

#### Clinical Data

Trained clinical researchers assessed OCD severity using the Yale-Brown Obsessive Compulsive Scale (Y-BOCS) and OCD subtype using the related Y-BOCS Symptom Checklist (Y-BOCS-SC) [16], in combination with an expert’s opinion. They measured anxiety and depression symptoms with the Hamilton Rating Scales for anxiety [17] and depression [18]. Finally, the sense of presence was measured with the Igroup presence questionnaire [19].

#### VR Game

The setup of the VR game is described and illustrated in detail in our pilot study [7]. In short, it concerned a first-person–perspective video game based in a house with 15 OCD-related events. Participants walked a set route through the house and were confronted with all these events in a preset order. At every event, after confrontation, participants were asked if they wanted to correct and then check an event, or if they wanted to proceed to the next event without intervening. Checks could be repeated as often as desired. At each event, participants rated their emotional responses including anxiety, tension, uncertainty, and urge to control on a digital 0-10 visual analog scale (VAS) after confrontation, correction, and checking. The VR game’s output scores included the VAS scores and the number of virtual compulsive actions performed.

For this study, we edited the output data of the VR game to allow communication with the fMRI scanner. After 35 minutes, both the game and scanning process were automatically stopped. Participants could see the white projection screen behind the head through a mirror fixed at a 45° angle to the head coil (standard MRI equipment). The VR game provides an immersive virtual reality “feel” because it has a first-person perspective and is projected close to the eyes in the scanner. The participant operated the VR game by means of 2 manual button boxes.

#### Acquisition of Images and Preprocessing

MRI scanning was performed using a 3.0T MRI scanner (Philips) using a 32-channel SENSE head coil. Scanning included a high-resolution T_1_-weighted structural scan for anatomical reference (repetition time=6.9 milliseconds, echo time=3.1 milliseconds, voxel size=1.20 mm isotropic, flip angle=8°, and 150 transverse slices). Additionally, at least 496 (range 496-883) BOLD scans were acquired using a T_2_*-weighted gradient multiecho echoplanar imaging sequence [20], with the following parameters: repetition time=2375 milliseconds, echo time=9/26.4/43.8 milliseconds, flip angle=76.1°, field of view=224 × 224 × 122 mm^3^, voxel size=2.8 × 2.8 × 3.0 mm^3^, matrix size=76 × 73, slice thickness=3 mm, slice gap=0.3 mm, number of slices=37, acquired in foot-head order. There was a maximum time frame of 35 minutes for playing the VR game inside the scanner.

We performed imaging analysis using Statistical Parametric Mapping (version 12; Wellcome Trust Centre for Neuroimaging). Data preprocessing consisted of realignment of images with respect to the middle volume, slice timing correction, coregistration of echoplanar imaging data to structural T_1_ data, normalization to Montreal Neurological Institute space (3 mm isotropic), and spatial smoothing using an 6-mm full width at half maximum Gaussian kernel. We checked for motion artifacts; for a patient with OCD, we had to omit the final 25% of the VR game scans, due to excessive motion artifacts (ie, >5-mm framewise displacement).

### Data Processing and Statistical Analysis

#### Clinical and VR Game Data

Demographic, clinical, and VR game data were analyzed using SPSS (version 26; IBM Corp). The VAS score of each emotional response upon confrontation with an OCD-related item was averaged for the 15 items. We performed Bonferroni correction to correct for testing of multiple emotional responses. Because of the small sample size, nonparametric tests were used to compare patients with healthy controls. We used the Mann-Whitney *U* test to compare continuous data (age and emotional responses) and Fisher exact tests for comparing categorical data (sex, nationality, schooling, and number of compulsions), including categorized questionnaire scores, because original scores did not qualify as continuous due to their limited distribution. Furthermore, in the group of patients with OCD, we calculated the reduction in emotional responses by subtracting the VAS score after the last compulsive action from the VAS score at confrontation. We used a 1-sample Wilcoxon signed rank test to assess the reduction in emotional responses after performing compulsive actions. The α value was set at .05 for significance.

#### Neuroimaging Data

**Figure 3:**
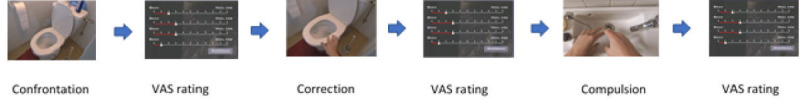
Events in the virtual reality game. VAS: visual analog scale.

Functional MRI data were analyzed using Statistical Parametric Mapping software (version 12) [21]. We performed individual subject analyses within the context of the general linear model, using delta functions convolved with a canonical hemodynamic response function to model events of interest. To enable this first-level analysis, we subdivided the events in the game in confrontation, correction, checking and, VAS rating events (Figure 3), which were contrasted with short time frames in the game during which no specific events took place. This resulted in a total of 10 regressors.

On the second-level between-group comparison, we conducted an independent samples *t* test to determine whether the OCD-related events in the VR game influenced brain activation differently between patients with OCD and healthy controls. We investigated group interactions using a priori regions of interest (ROIs). We defined the bilateral amygdala, OFC, ACC, right putamen, and right insula as a priori ROIs. We used a threshold of 0.01 (0.05 divided by 5; corrected for multiple ROIs) for significance. To accommodate the low statistical power due to the small sample size, we also reported results at a trend significance level with a threshold of *P*<.10. We corrected for multiple comparisons at the voxel level (family-wise error) using a small-volume correction for ROIs, which were based on the automatic anatomical labeling atlas [22], using the WFU Pickatlas tool [23]. To determine correlations of Y-BOCS scores with fMRI data in SPSS, we used Marsbar [24] to extract parameter estimates from the bilateral amygdala and right insula in patients with OCD. To determine correlations of the fMRI data with the Y-BOCS scores, the Spearman correlation coefficient was used.

## Results

### Demographic and Clinical Data

Demographic and clinical data of the study participants are shown in Table 1. Patients showed significantly more obsessive-compulsive and depressive symptoms; however, anxiety symptoms (assessed using the Hamilton Rating Scale for Anxiety) and mean scores of the Igroup Presence Questionnaire did not categorically differ significantly between patients and healthy controls (*P*=.21 and *P*=.17, respectively)*.* Patients with OCD had a mean Y-BOCS score of 23, which indicates moderate symptom severity. All patients had symptoms from multiple OCD dimensions. The most common dominant dimensions included perfectionism or symmetry for 44.4% (n=4) and taboo thoughts (aggressive or sexual intrusions) for 33.3% (n=3) of patients with OCD. Five patients with OCD were treated with a selective serotonin reuptake inhibitor (SSRI) or serotonin and norepinephrine reuptake inhibitor (SNRI), and 4 patients were unmedicated. Four patients experienced comorbid disorders including generalized anxiety disorder, depression, and social anxiety.

**Table 1:** Demographic and clinical data of patients with obsessive-compulsive disorder and healthy controls.

Characteristics	Patients (n=9)	Controls (n=9)	*P* value
Age (years), mean (SD)	29 (8.0)	29 (8.5)	.97
Male sex, n (%)	4 (44)	4 (44)	>.99
Dutch nationality, n (%)	8 (89)	9 (100)	>.99
Tertiary education, n (%)	3 (33)	5 (56)	.64
HAM-A^a^ score, mean (SD)	12 (7.5)	1 (2.0)	.21
HAM-D^b^, mean (SD)	11 (4.5)	1 (2.1)	.002
Y-BOCS^c^ score, mean (SD)	23 (2.8)	0 (0)	<.001
IPQ^d^ score, mean (SD)	1.97 (0.96)	1.53 (0.75)	.17

^a^HAM-A: Hamilton Rating Scale for Anxiety.

^b^HAM-D: Hamilton Rating Scale for Depression.

^c^Y-BOCS: Yale-Brown Obsessive Compulsive Scale.

^d^IPQ: Igroup Presence Questionnaire.

### VR Game Data

The provoked emotional responses when playing the VR game are shown in Table 2. These represent the difference in mean VAS scores at confrontation over all 15 items and VAS scores at the baseline measurement. Playing the VR game provoked significantly more anxiety, but not tension, uncertainty, and an urge to control in patients with OCD compared to healthy controls. Furthermore, patients with OCD showed significantly more compulsive behavior in the VR game than healthy controls; patients performed a mean of 0.46 (SE 0.14) compulsions per event, healthy controls performed a mean of 0.07 (SE 0.02) compulsions per event (*P*=.03). Finally, in patients with OCD, we found a significant reduction in anxiety (mean 1.54, SE 0.60; *P*=.001), unrest (mean 3.09, SE 0.77; *P*=.008), and uncertainty (mean 1.72, SE 0.61; *P*=.01) but not in the urge to control (mean 2.16, SE 0.86; *P*=.02) after performing virtual compulsive actions.

**Table 2:** Provoked emotional responses during the virtual reality game measured using the visual analog scale (VAS).

	VAS scores of patients with obsessive-compulsive disorder (n=9), mean (SE)	VAS scores of controls (n=9), mean (SE)	*U* value	*P* value
Change^a^ in anxiety	1.01 (0.38)	0.13 (0.30)	12.0	.01
Change in tension	0.78 (0.72)	0.21 (0.35)	36.0	.73
Change in uncertainty	1.47 (0.57)	–0.03 (0.19)	19.0	.06
Change in the urge to control	1.58 (0.63)	1.31 (0.37)	38.0	.86

^a^Difference between scores at baseline and at confrontation.

### Neuroimaging Data

**Figure 4:**
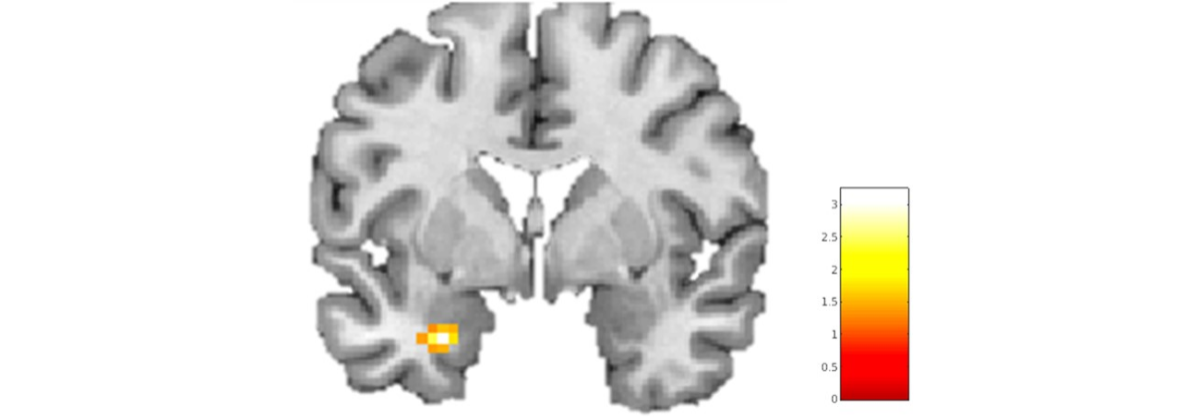
Results of analysis of regions of interest. Trend significant cluster of hyperactivation in the left amygdala in patients with obsessive-compulsive disorder (OCD) compared with healthy controls during confrontation with OCD-related events. Montreal Neurological Institute coordinate: y=–4.

**Figure 5:**
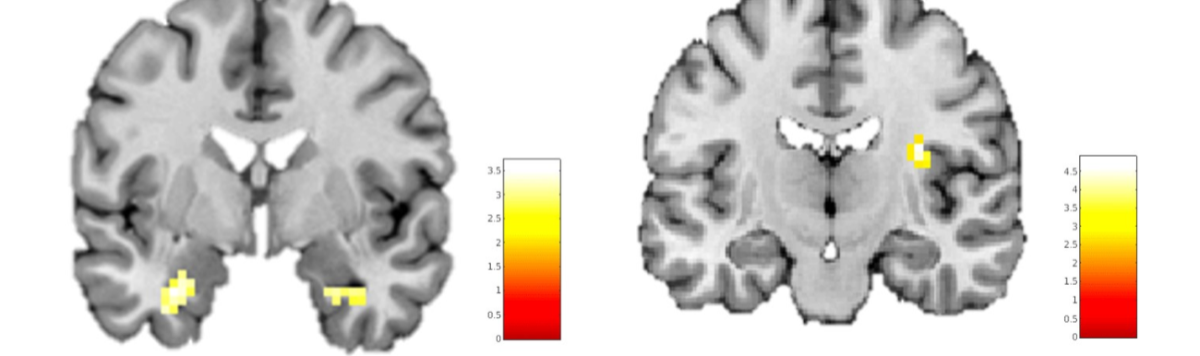
Results of analysis of regions of interest. Trend significant clusters of hyperactivation in patients with obsessive-compulsive disorder compared with healthy controls during performance of virtual compulsive actions, in the left and right amygdala (A; Montreal Neurological Institute [MNI] coordinate: y=–4) and right insula (B; MNI coordinate: y=–19).

Since 4 patients were unable to finish the VR game within 35 minutes, patients with OCD were exposed to a mean total number of 13.89 (SD 1.27) events during the VR game scan, whereas all healthy controls were exposed to all 15 (SD 0) events. After correction for multiple comparisons, there were no significant differences in brain activation between patients with OCD and healthy controls when playing the VR game. However, during confrontation with the OCD-related events in the game, a larger increase in left amygdala activity was observed in patients with OCD than in healthy controls at the trend level (Figure 4; peak voxels: x, y, and z=–27, –4, and –25; T=3.24; *P*_fwe,svc_=.07). When performing the virtual compulsive actions, a larger increase in left amygdala (Figure 5A; peak voxels: x, y, and z=–27, –4, and –22; T=3.61; *P*_fwe,scv_=.06), right amygdala (Figure 5A; peak voxels: x, y, and z=30, –4, and –28; T=3.27; *P*_fwe,svc_=.09), and right insula (Figure 5B; peak voxels: x, y, and z=33, –19, and 20; T=4.61; *P*_fwe,svc_=.09) activity was observed in patients with OCD than in healthy controls at trend level. Task-related activity in the ROIs OFC, ACC, and right putamen was not significantly increased in patients with OCD compared to that in healthy controls. Finally, healthy controls showed no areas of increased BOLD response upon confrontation with OCD-related events or when performing virtual compulsive actions compared to patients with OCD.

### Correlation Between Y-BOCS Scores and Brain Activation

Based on the aforementioned results, we focused on the correlation between amygdala and insula activation and Y-BOCS scores in patients with OCD. Upon confrontation with OCD-related items, there was no significant correlation between left amygdala activation and the Y-BOCS score (*r_s_=–*0.542, *P*=.13). When performing virtual compulsive actions, there were no significant correlations between left amygdala (*r_s_*=–0.192, *P*=.65) or right amygdala (*r_s_*=–0.419, *P*=.30) and right insula (*r_s_*=–0.467, *P*=.24) activation and Y-BOCS scores.

## Discussion

### Principal Findings

In this study, we performed an fMRI scanning session during a VR game for OCD, allowing us to examine brain regions upon confrontation with virtual symptom-provoking events and compulsions. We replicated findings from our previous study, showing that this VR game provoked more anxiety and virtual compulsive actions in patients with OCD than in healthy controls [6]. In the patient group, we also found a decrease in negative emotions following compulsive actions. Our results confirm that the VR game can provoke anxiety and virtual compulsions, which modulate negative emotions. We found no significant differences in brain activation between patients with OCD and healthy controls. The results show increased activity in the bilateral amygdala and the right insula at the trend level. We found an increase in left amygdala activity upon confrontation and an increase in bilateral amygdala and right insula activity with compulsive actions in patients with OCD. Contrary to our hypothesis, we did not find differences in brain activity in the OFC, ACC, and right putamen.

### Comparison to Prior Work

The increase in left amygdala activity in response to confrontation is in line with previous symptom provocation studies [9]. The amygdala is involved in the detection of salient events and the mediation of negative emotions such as fear and anxiety [25,26]. The activity is associated with increased attention toward events and provoked feelings of anxiety. The laterality of the amygdala’s response may be explained by fear modulation of the left amygdala in response to learned, subject-dependent, aversive stimuli, in contrast to fear modulation by the right amygdala in generally aversive stimuli [27]. Increased bilateral amygdala and right insula activity during virtual compulsive actions is in line with previous provocation studies [9]. In our study, we observed brain activity while participants actually performed virtual compulsions. This is unique, since other studies use pictures, emotional faces, or written words to provoke symptoms. Our approach is a good example of an ecologically valid experiment that shows engagement of the amygdala and insula.

Thorsen et al [9] found pronounced right insula activation in studies with comorbid anxiety or mood disorders. In another study, the right insula was activated in response to disgust-inducing pictures in patients with contamination fear [28]. Indeed, the insula is suggested to play a role in processing disgust, and, in particular, the contamination/washing dimension of OCD is associated with higher disgust sensitivity [29]. Furthermore, Luigjes et al [30] found increased insula activation during risk processing in risk-averse patients with OCD, mainly in those with the doubt/checking dimension of OCD. The disgust and high risk–related virtual compulsions in our VR game (eg, washing hands after touching a dirty toilet or turning off a running flat iron) could have contributed to right insula activation.

In contrast to former studies, we did not find a difference in activity in the OFC or ACC. Most neuroimaging symptom provocation studies found increased activity in the OFC and ACC [9,11]. In one study, hypoactivation of the left ACC was observed in response to a handshake from a dirty virtual avatar in patients with OCD [31]. Furthermore, we did not find a correlation between the degree of brain activation and severity of symptoms. These results are nevertheless consistent with those of the meta-analysis of Thorsen et al [9]; the latter did not find a correlation between amygdala or insula activity and symptom severity.

### Limitations

Our study has a few limitations. First, since this is a proof-of-concept study, we decided to recruit a small sample of 9 patients with OCD and 9 healthy controls, leading to limited statistical power. This impedes drawing definite conclusions regarding the ability of the VR game to activate the OCD-related neural regions in patients compared to healthy controls. This could also explain why only trend-level activation patterns were observed in the amygdala and insula.

Second, in our group of patients with OCD, 5 patients used a SSRI or SNRI and 4 experienced a comorbid anxiety or mood disorder. Thorsen et al [9] found a negative correlation between SSRI use and right amygdala activation, and more pronounced right amygdala and less pronounced left amygdala activation in studies with more comorbid anxiety and mood disorders. Hence, in our results, both medication use and comorbid disorders could have affected left and right amygdala activation. Third, we used the “neutral” scenes (eg, the camera turning toward a wall) as contrast in the analyses during the game, and we cannot exclude the premise that participants already anticipated new events during the neutral scenes. If anything, this would have led to less pronounced activation patterns in the OCD-related brain regions in response to OCD-related events than to neutral events. Finally, 4 out of 9 patients with OCD indicated that their specific obsessions and compulsions were not triggered by the VR game. This indicates that the VR game, despite its comprehensive design, is not able to trigger OCD in all patients, possibly because the VR game did not represent all OCD dimensions. Indeed, studies have found distinct patterns of brain activation with OCD dimension–specific picture sets [32] or fully individualized picture sets [8].

### Conclusions

In this proof-of-concept study, the VR game activated the bilateral amygdala and right insula at the trend level in patients with OCD, especially when performing virtual compulsions. Since this was a proof-of-principle study with 9 patients, it is important to replicate these results in studies with a larger sample size. Our results suggest that immersive symptom provocation, with the possibility to conduct virtual compulsive actions, may allow us to study brain regions in patients with OCD in a more ecologically valid context, and, as such, can be seen as a stepping stone toward more research in this area. In particular, the possibility to observe brain activation when performing virtual compulsive actions might teach us more about the involved brain regions in this complex process that has proved difficult to study in an MRI scanner. So far, the VR game was not able to activate the whole OCD circuit including the OFC and ACC, possibly due to limited power because of the small sample size.

To our knowledge, this is the first study among patients with OCD using an innovative and interactive VR game entailing multiple OCD dimensions for symptom provocation inside a MRI scanner. New possibilities arise, for example, with the development of MRI-suitable head-mounted displays, to gain even higher levels of presence and immersion inside the MRI scanner. Furthermore, new VR technology allows for personalization of the virtual environment to the OCD dimension, which may improve power to detect brain regions [8]. Further research with larger sample sizes is needed to determine whether using a virtual environment on a head-mounted display that can be adjusted to OCD subtypes will lead to increased activation of neural regions related to OCD. If activation of OCD-related neural regions can be achieved during confrontations with events and while performing compulsive actions in a VR environment, this suggests the feasibility of exploring the neural basis of near–real-life OCD symptoms. This approach could yield deeper insights into the complex pathological foundations of OCD.

## Abbreviations

ACC: anterior cingulate cortex
BOLD: blood oxygenation level–dependent
DSM-IV: Diagnostic and Statistical Manual of Mental Disorders, Fourth Edition
fMRI: functional magnetic resonance imaging
MRI: magnetic resonance imaging
OCD: obsessive-compulsive disorder
OFC: orbitofrontal cortex
ROI: region of interest
SNRI: serotonin and norepinephrine reuptake inhibitor
SSRI: selective serotonin reuptake inhibitor
VAS: visual analog scale
VR: virtual reality
Y-BOCS: Yale-Brown Obsessive Compulsive Scale
Y-BOCS-SC: Yale-Brown Obsessive Compulsive Scale Symptom Checklist

## Data Availability

The data sets generated or analyzed during this study are available from the corresponding author on reasonable request.
